# Classification tree analysis of second neoplasms in survivors of childhood cancer

**DOI:** 10.1186/1471-2407-7-27

**Published:** 2007-02-02

**Authors:** Janez Jazbec, Ljupčo Todorovski, Berta Jereb

**Affiliations:** 1 Division of oncology and hematology, Department of Pediatrics, Medical Center, Vrazov trg 1, Ljubljana, Slovenia; 2 Institute Josef Stefan, Jamova 39, Ljubljana, Slovenia; 3 Institute of Oncology, Zaloška 2, Ljubljana, Slovenia

## Abstract

**Background:**

Reports on childhood cancer survivors estimated cumulative probability of developing secondary neoplasms vary from 3,3% to 25% at 25 years from diagnosis, and the risk of developing another cancer to several times greater than in the general population.

**Methods:**

In our retrospective study, we have used the classification tree multivariate method on a group of 849 first cancer survivors, to identify childhood cancer patients with the greatest risk for development of secondary neoplasms.

**Results:**

In observed group of patients, 34 develop secondary neoplasm after treatment of primary cancer. Analysis of parameters present at the treatment of first cancer, exposed two groups of patients at the special risk for secondary neoplasm. First are female patients treated for Hodgkin's disease at the age between 10 and 15 years, whose treatment included radiotherapy. Second group at special risk were male patients with acute lymphoblastic leukemia who were treated at the age between 4,6 and 6,6 years of age.

**Conclusion:**

The risk groups identified in our study are similar to the results of studies that used more conventional approaches. Usefulness of our approach in study of occurrence of second neoplasms should be confirmed in larger sample study, but user friendly presentation of results makes it attractive for further studies.

## Background

As the number of childhood cancer survivors grows and the period of follow-up lengthens, increasing attention is directed towards the delayed adverse effects of therapy. The late effects of treatment on many organs have been described. These include the, cardiovascular, skeletal, endocrine, dental, hepatic, pulmonary and renal systems. Psychosocial, educational and neuropsychological problems are also common, but among the most serious of the delayed complications is the appearance of second neoplasms (SN). The better the treatment results become for the primary malignancy, the more may long-term results be compromised by secondary cancers [1]. Relevant reports of the cumulative probability of developing SN vary from 3.3% to 25% at 25 years from diagnosis. Thus, the risk of developing another cancer can be up to 35 times greater than in the general population [2].

SNs develop after interaction among many independent factors to which the patient is exposed before, during and after treatment of the first malignancy. Some of those factors may have synergistic oncogenic effects on the development of SNs, and design of prospective studies to identify those risk factors is difficult, due to the long latency period. In our retrospective study, we have used the decision tree multivariate method to identify the group of childhood cancer patient with the greatest risk for development of SN.

## Methods

### Patients

The study included 1577 cancer patients younger than 16 years of age registered at the Cancer Registry of Slovenia in the period from 1-1-1961 to 12-10-2000. The decision tree analysis was performed on a group of 849 first cancer survivors, among whom 34 developed a SN. An SN was defined as a malignant neoplasm in a new location that was neither the result of direct spread nor a metastasis from the primary neoplasm. Also included among the SNs was a neoplasm in the same location as the primary but of different histological type [3]. Primary neoplasms were categorized according to histology as: leukemia, Hodgkin's disease, non-Hodgkin lymphoma, Ewing sarcoma, osteogenic tumors, nephroblastoma, neuroblastoma, hepatoblastoma, rhabdomyosarcoma, retinoblastoma, thyroid cancer, germ-cell tumors, tumors of central nervous system (CNS) and others. The group of "others" consisted of carcinomas of different organ systems in 41 cases and two melanomas. They were grouped together because each particular group was too small for further analysis. Data in the database included patient's name, sex, date of birth, clinical diagnosis, histologic type of the neoplasm, date of the diagnosis, treatment modality, date and status at the last follow-up. Detailed information on chemotherapy and radiotherapy was not included in the database. Table 1 presents the independent and dependent variables used for multivariate analysis.

All data were collected through the childhood cancer follow-up program in Slovenia. One pediatric-oncology center in the Department of Pediatrics, University Medical Center, Ljubljana, serves as a national referral center for all pediatric patients with malignant diseases. It covers the population of Slovenia that approximates 2 million. After the end of treatment all children are followed in the center until the end of schooling or for at least four years. After that, they are followed at the outpatient Clinic for Late Effects at the Institute of Oncology. A team there, headed by an oncologist known to the patient as a member of the pediatric follow-up team, continues follow-up for life [4]. Fewer than 5% of patients were lost to follow-up because of permanent migration outside the territory of the Republic Slovenia. All of them were treated before 1990.

The study was performed in compliance with the Helsinki Declaration with the approval N° 38/11/96 of National Medical Ethics Committee of Slovenia

### Classification tree analysis

Classification tree is a method for multivariate analysis that allow for study of simultaneous influence of a series of independent variables on the one dependent variable. The analysis is performed by successive divisions of the original group of cases into pairs of subgroups, where each division is based on the value of a single independent variable. The variable that produces most pure pair of case subgroups is chosen for a division (division being often referred to as a split). A purity of a case group is measured as a fraction of cases with the same value of the dependent variable: a completely pure group contains cases that have the same outcome. Each of subgroup in the pair becomes a parent group in the next step of the analysis and is therefore further divided in the same way. The division of cases stops when the group of cases is completely pure or when it contains less than operator-defined minimal number of cases. In our study, the C4.5 [5] program for building classification trees was used. C4.5 allows the setting of several parameters that influence the branching and quality of final classification tree: most notably there is one parameter that determines the smallest number of cases to be included in a single group (mentioned already above), and another parameter that determines the degree of post-pruning performed. For details please refer to the description in [5]. The optimal values of these parameters were determined using a standard cross-validation method [6-8]. Following this method, we systematically try different combinations of parameter settings and use cross-validation to estimate the performance of the tree on unseen cases, and choose the settings that lead to the best tree performance. Using these optimal settings, we build a tree that is then used in further analysis and present in next section. We tried 5 possible values for the minimal number of cases in a group parameter (from 1 to 5) and 7 possible values for post-pruning confidence (1, 5, 10, 25, 50, 75, and 99%), which lead to 5 × 7 = 35 possible parameter settings.

The usual performance measure for classification trees is the accuracy of the tree when predicting the outcome (the value of the dependent variable) on samples not seen during the process of tree building. Note however, that since the SN has been observed in only a minority of patients (about 4%), the classification tree algorithm tend to build a single group of cases that classify all the patients as non-SN cases, this simple tree have a prediction accuracy of 96% that can not be significantly improved. This tree however misclassifies all the patients where SN is observed as non-SN cases. Note that this misclassification is much more serious for the patient when the opposite one, where a non-SN patient is predicted to have SN. The tool to deal with this issue in classification trees is to assign different costs to misclassifications, i.e., specifying that misclassifying a SN patient as a non-SN case is X time worse (or more costly) than the misclassifying a patient in opposite direction, where X is a user-specified parameter. In business applications of classification trees, misclassifications can be easily related to costs and these can be then used to estimate the X parameter setting. However, in our case, this is non-trivial issue: we know that this X is larger than 1. Thus, we approach this problem using the cross-validation procedure outlined above: we use it to find optimal settings for the X parameter. We choose the parameter that lead to minimal number of misclassifications of a SN patient as a non-SN case. In the experiments with C4.5, we increase the cost of this misclassification type using 7 different settings, starting with the default one of 1:1 (equal costs of both misclassifications), through 1:5, 1:10, 1:25, 1:50, 1:100, and 1:200.

Note finally, that since we use an alternative performance criterion, the classification tree obtained the cross-validation procedure outlined above is not expected to provide accurate classification of cases into SN and non-SN classes. Instead of using the tree as an accurate predictor, we are interested in analyzing the tree structure and identifying the risk groups where incidence of SN is significantly higher than the one observed in the whole population of 849 cancer survivors included in the study.

## Results

Highly branched tree, where most of the cases are misclassified, may be result of low rate of events or low predictability of the factors used to develop the tree. In the analysis of the entire group of 1577 childhood cancer patients, the number of SN cases is below 3% and all the SN cases were classified as non-SN cases. Therefore we reduced our analysis on the group of children who survived their first cancer. There were 849 patients in this group and 34 developed SN. We have build several classification tree models with different misclassification costs in the algorithm. We considered misclassification of an SN case in the group without SN as a more severe mistake than vice versa. In the extreme case, with misclassification cost 5:1, we have built a tree where all SN cases were allocated in the group without SN. On the other side, if the misclassification cost was set too high, there were too many cases without SN classified as patients with SN. Table 2 presents a sample of classification results obtained using three different parameter settings. The table includes results for the optimal parameter setting, where the misclassification cost value was set to 25:1, post-pruning confidence value to 50% and minimal number of cases in a group to 2.

**Figure 1:**
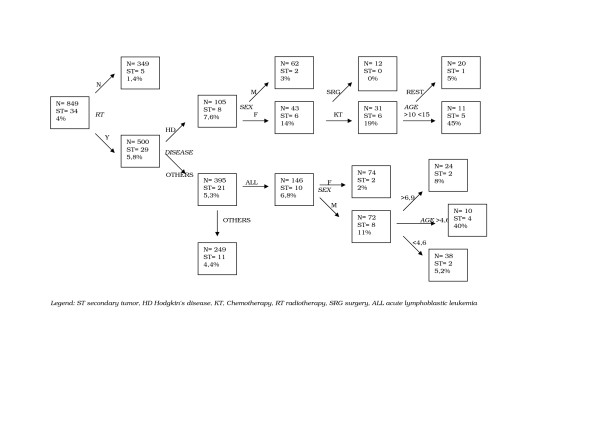
Clasification tree for the risk of secondary neoplasm after treatment for childhood cancer. Analysis of 849 childhood cancer survivors from Cancer Registry of Slovenia.

On the basis of these results presented in Table 2, we were able to choose the parameter setting that gave the lowest number of SN cases being misclassified as non-SN cases. Figure 1 depicts the classification tree obtained using this parameter setting.

Despite the optimal setting, branching of the tree is still considerable. There are many sets with individual SN cases and some clusters in which misclassified non-SN cases predominate. In the graphic presentation of the pruned tree the first factor that divides our cohort is radiotherapy. In the group of patients treated without radiotherapy, only 1,4% patients developed SN, which is considerable less than in the group of irradiated patients (5,8%). From this point we can follow two paths. The first one encompasses patients with Hodgkin's disease. At the end of the non-Hodgkin's disease branch, a group of females, aged between 10 and 15 years at first diagnosis and treated with chemotherapy, can be identified as a group in which the risk of an SN reaches 45%. The other path reveals a group of male patients with acute leukemia, who were aged between 4.6 and 6.6 years of diagnosis of leukemia. In these groups the risk for SN reaches 40%. Both incidence rates of 40% and 45%, observed in these groups of patients, are significantly higher compared to the observed 4% incidence in the whole population. The fact that can be easily confirmed using a simple ChiSquared test, see Table 3 for results.

## Discussion and conclusion

In general the estimation of risk varies, between hospital based and population based studies [2], probably due to more complete follow-up in the former registries. Our population based study, differs from similar studies also for defining a period at risk for SMN, from the diagnosis of primary cancer on. Varying cure rates in different time periods also have impact on estimated risk. The period covered in our study starts in early seventies, when cure rate of childhood cancer was still very low.

In our study of 849 childhood cancer survivors we have performed a multivariate analysis using classification trees to identify groups that are at special risk for the development of a SN. The group at highest risk was identified as girls with Hodgkin's disease, aged between 10 and 16 years at first diagnosis, who were treated with combined of chemo- and radio-therapy. In all of this cases, the SN was a carcinoma, with the latent period ranging from 3 to 16.5 years after treatment of the Hodgkin's disease. These results are similar to the observation of Beaty et. al. [9], who found statistically significant higher risk for SN in adolescents treated for Hodgkin's disease. Bhatia and coworkers found 6.7 fold higher risk for SN in patients treated for Hodgkin's disease between 10 and 16 years of age [10]. They also found the risk for secondary solid tumors after a combination of chemotherapy and radiotherapy to be twice as great as after chemotherapy without radiotherapy. It is possible that some tissues are particularly vulnerable to the carcinogenic effect of chemotherapy and radiotherapy during puberty.

The challenge is to maintain the high rate of cure in Hodgkin's disease and at the same time reduce the risk for second malignancies. Some modern protocols of treatment of Hodkin's disease have already reduced or completely omitted radiotherapy for patients with low stages of disease. Löning et. al found radiation therapy as significant risk factor for SN after treatment of childhood acute lymphoblastic leukemia [11]. This is in contrast with the results of Dalton et. al. [12]. Löning also states that particularly young children are at increased risk when irradiation has been used. Intensive chemotherapy regimens do not predict a higher risk as reported in several studies [13,14]. In the Childhood Cancer Survivor Study, the diagnosis of leukemia was independently associated with the occurrence of a second malignant tumor of the central nervous system, as was younger age at diagnosis [15].

The improved survival rate of children with cancer should not be overshadowed by the incidence of SNs. Nonetheless, patients and health care providers should be aware of the populations at greatest risk for this serious complication, and focus their efforts on primary and secondary prevention in this vulnerable population. Using the C 4.5 algorithm for building classification trees, we were able to construct subgroups at different risk, by logical combination of patients characteristics. The risk groups identified in our study are similar to the results of studies that used more conventional approaches. In contrast to traditional regression methods (e.g. Cox proportional hazard regression) which compute prognostic index as a weighted average of patients' characteristics, in the classification tree model the subgroups are based directly on the patients' characteristics. The model shows the correlation between the various independent variables and its influence on the end result [16]. Another advantage of the method is in its simple and intuitive nature (i.e. find the best split by examining all possible splits in all available variables, form subgroups based on this split, repeat in all subgroups) [17].

Classification trees have been used in medical and health care applications for more than 20 years and have been shown to be a powerful classification tool in various areas [18]. In oncology the method has been used for tumor classification, evaluation of biomarkers [19-23]. The sample size represents a limitation in our study, but the method used is a potentially powerful tool for investigating multilevel interactions [24]. Occurrence of secondary neoplasms may well be the result of complex interactions of several independent factors such as genetic predisposition, treatment related factors and environmental exposures. The approach to the analyses of a larger sample here described might serve to validate the technique.

## Competing interests

The author(s) declare that they have no competing interests.

## Authors' contributions

All authors read and approved the final manuscript. JJ carried out the patient recruitment, acquisition and interpretation of the data. He also drafted the manuscript. LD performed the statistical analysis and drafted the manuscript. BJ is a leader of Late effect study group and participated in the design of the study, carried out the patient recruitment and gave final approval of the version to be published.

## Pre-publication history

The pre-publication history for this paper can be accessed here:



## Figures and Tables

**Table 1:** Description and values of the independent variables and the dependent variables (last row) used for multivariate analysis.

**Variable name**	**Description**
Sex	male (485), female (346)
age_at_diagnosis	numeric
histology_type (categories)	leukemia, Hodgkin's disease, non-Hodgkin lymphoma, Ewing sarcoma, osteogenic tumors, nephroblastoma, neuroblastoma, hepatoblastoma, rhabdomyosarcoma, retinoblastoma, thyroid cancer, germ-cell tumors, tumors of central nervous system, others
Surgery	yes (481), no (368)
Radiotherapy	yes (500), no (349)
Chemotherapy	yes (598), no (251)
second_neoplasm	yes (34), no (815)

**Table 2:** Comparison of classification results obtained using cross-validation for three different C4.5 parameter settings.

**Misclassification cost setting 5:1, post-pruning confidence 1%**		
	Classified as	
Observed	SN	Non-SN
SN	0	34
Non-SN	0	815

**Misclassification cost setting 10:1, post-pruning confidence 1%**		

	Classified as	
Observed	SN	Non-SN
SN	8	26
Non-SN	66	749

**Misclassification cost setting 25:1, post-pruning confidence 50%**		

	Classified as	
Observed	SN	Non-SN
SN	31	3
Non-SN	18	797

The settings comprise of three different misclassification costs, two different post-pruning confidence values, and the default value of 2 for the minimal number of cases in a group parameter. In each table, the number of SN patients being misclassified as non-SN is typed in bold-face. To induce the final tree, presented in Figure 1, we selected a parameter setting that lead to minimal number of such misclassifications.

**Table 3:** Comparison of SN incidence in the two risk groups, identified using the classification tree from Figure 1, with the incidence of SN in the whole observed population

**First identified risk group (girls with Hodgkin's lymphoma): ChiSquared p-value is much smaller than 0.01 (in a range of 10**^-11^**)**			
**Group of patients**	**SN**	**Non-SN**	**Total**

Identified risk group	5	6	11
Others	29	809	838
All included in the study	34	815	849

**Second identified risk group (boys with acute lymphoblastic leukemia): ChiSquared p-value is much smaller than 0.01 (in a range of 10**^-9^**)**			

**Group of patients**	**SN**	**Non-SN**	**Total**

Identified risk group	4	6	10
Others	30	809	838
All included in the study	34	815	849

ChiSquared test show significant difference in both cases.
